# The Semiosis of “Side Effects” in Genetic Interventions

**DOI:** 10.1007/s12304-016-9274-3

**Published:** 2016-10-15

**Authors:** Ramsey Affifi

**Affiliations:** University of Edinburgh, Edinburgh, UK

**Keywords:** Heterarchy, Multi-level semiosis, Bateson, Genetic engineering, Side effects, Extended evolutionary synthesis, Phenotypic plasticity, Niche construction

## Abstract

Genetic interventions, which include transgenic engineering, gene editing, and other forms of genome modification aimed at altering the information “in” the genetic code, are rapidly increasing in power and scale. Biosemiotics offers unique tools for understanding the nature, risks, scope, and prospects of such technologies, though few in the community have turned their attention specifically in this direction. Bruni (2003, 2008) is an important exception. In this paper, I examine how we frame the concept of “side effects” that result from genetic interventions and how the concept stands up to current perspectives of the role of organism activity in development. I propose that once the role of living systems in constructing and modifying the informational value of their various developmental resources is taken into account, the concept of a “side effect” will need to be significantly revised. Far from merely a disturbance brought about in a senseless albeit complex system, a biosemiotic view would take “side effects” as at least sometimes the organism’s active re-organization in order to accommodate or make use of novelty. This insight is nascent in the work of developmental plasticity and niche construction theory (West-Eberhard 2003; Odling-Smee et al. 2003), but it is brought into sharper focus by the explicitly interpretive perspective offered by biosemiotics. Understanding the “side effects” of genetic interventions depends in part on being able to articulate when and where unexpected consequences are a result of semiotic activity at various levels within the system. While a semiotic interpretation of “side effects” puts into question the naive attitude that would see all unintended side effects as indications of disturbance in system functionality, it certainly does not imply that such side effects are of no concern for the viability of the organisms in the system. As we shall see, the fact that such interventions do not respect the translation of information that occurs in multi-level biological systems ensures that disruption is still likely. But it does unprivilege the human agent as the sole generator of meaning and information in the products of biotechnology, with important consequences on how we understand our relationship with other species.

## Introduction

Proponents of genetic engineering tend to minimize the likelihood of “side effects” and insist that those that do occur can be attenuated, controlled for, or bred out of the system (ex. Butaye et al. 2005; Kohli et al. 2004). Critics often hold the view that any genetic intervention necessarily corrupts the interconnectedness of the genome and that the “side effect” is evidence of systemic disruption (see Ho (2014)). This paper proposes that the main proponents and critics of genetic interventions both misunderstand the nature of the “side effects” that often occur as a result of the intervention. The insufficiency of these interpretations of “side effects” is already hinted at in contemporary approaches to understanding development and evolution that focus on the active causal role of organisms in both processes. For instance, niche construction theory (Odling-Smee et al. 2003) and developmental plasticity (West-Eberhard 2003, 2005) seem to imply that a “side effect” may be (at least in part) an accommodation or transformation of the intervention and its products, an actively constructed result of the organism striving to convert the modification into some functionality. As we shall see, biosemiotics is consonant with these approaches but pushes further by explicitly recognizing that much of plasticity and niche construction is driven by the interpretive activities of goal-based and meaning making organisms and their organismic subsystems. If this is the case, then it is unavoidable that we will find that at least some of the “side effects” of genetic interventions across various biological levels from the chromosome to the ecosystem, may well be interpretive responses to the changed conditions directly presented by the genetic intervention or by some later downstream consequence of the intervention. Of course, an interpretive response is not necessarily “beneficial” to the biological systems involved, so this qualification of the term “side effect” in no way entails obviating the need for careful study and assessment of genetic interventions. This is particularly true when considering the nature of the intervention into the multi-level chains of information translation between genomes and ecosystems. But it does indicate that the organism is an active player in what and how information arises for it, and that information cannot be solely dictated by the human intervening in the system. It also indicates that the way the organism will be interpreting edited genes, transgenes, and their products is itself contingent upon broader environmental contexts that it finds itself in.

This paper has the following structure. First, I describe how several branches of modern evolutionary theory (collectively under the term “extended evolutionary synthesis”) present the view of organisms as active, interpretive agents in the course of their own development and evolution. I then show how biosemiotics can converge fruitfully with these branches by helping us take direct account of meaningful organism-directed activity. In part, this involves understanding how organismal semiosis is hierarchical (or better, “heterarchical”), such that the interpretive activities of organisms at one level are themselves interpreted and responded to elsewhere in the system in ways that often involve the summing, silencing, and translating of information. The nature of these processes suggests that new sorts of disturbances may more easily and more often occur as a result of genetic interventions. However, because interpreting genetic interventions at multiple dynamic levels across the system can involve co-opting the novelty into new functionality, some of the apparently disturbing side effects that occur may well be the result of the interpretive activity of semiotic agents at various loci. While I do not claim that all unintended side effects are functional semiotic interpretants, I do claim that it is essential to examine closely what, when and where integrative side effects occur as a result of semiosis, and to contrast these with cases where the disruptive side effects compromise semiosis and system functionality. I end the paper by also arguing that our capacity to understand the semiosis of “side effects” has important implications with respect to how we view the relationship between humans and the rest of the biosphere. A semiotically informed understanding of “side effects” acknowledges the active role that organisms play in ontogeny and challenges the view that humans are the sole interpreters of information and meaning.

## The Active Organism in the “Extended Evolutionary Synthesis”

The environmental and physical nature of biological systems, their developmental history, but also their activity all channel the way in which organisms develop. Although these are all sources of “developmental bias^1^“ (Arthur 2004) crucial for understanding the form and function of life, the latter factor, the active role of the organism as interpretive and integrative system, has not been sufficiently integrated into evolutionary theory -nor indeed into understanding modern biotechnology. A premise of this paper is that understanding biosemiosis is crucial for understanding developmental bias, which in turn provides insights into the developmental and ecological effects of genetic interventions. To see why this is the case, we must first examine some aspects of modern evolutionary and developmental biology that maintain the importance of understanding the active role of the organism in its own ontogenesis.

A convergence of approaches under the term “Extended Evolutionary Synthesis” (EES) (Pigliucci and Müller 2010) has already dislodged the idea that gene changes lead the process of evolution, and has placed the organization and behaviour of the developing phenotype as a central causal loci for understanding and integrating ecological and evolutionary change. EES includes a range of interconnected research programs defined by conventional disciplinary boundaries (such as various branches of evo-devo (West-Eberhard 2003; Kirschner and Gerhart 2005; Müller and Newman 2003), niche constructionism (Odling-Smee et al. 2003; Badyaev and Uller 2009), and multiple forms of inheritance (Jablonka and Lamb 2005; Gilbert et al. 2015)), all divisions that appear increasingly arbitrary given the complex and integrated multilevel nature of biological systems.^2^ According to Laland et al. (2015), EES retains the importance of classical evolutionary mechanisms such as Darwinian selection, populational thinking, and genetic inheritance but places these within a more general framework that allows for reciprocal causality between developmental elements and their environments at various levels. While these are positive developments, EES is motivated to understand organism organization and behaviour with the risk of falling back on adaptionist arguments that explain only that the *capacity for such changes* has been vetted by an earlier selection process.

At root in the EES amendment of classical evolutionary explanation is the fact that neither genetic, epigenetic, cellular, organismic, nor environmental change is random in the ways assumed by the Modern Synthesis (Fisher 1930; Huxley 1942; Mayr 1982). Change at each level is biased because either the organism or its organismic subcomponents (such as cells) directly construct their own developmental elements, and because ‘random’ elements get re-worked and often integrated functionally by the developing organism. Of course, it has long been known that functional behaviour emerges ontogenetically on some of these levels but the importance of development was downplayed. Development was considered to have no ultimate causal role in the emergence of form and novelty (Mayr 1961). In highlighting the importance of the organism as the locus for reciprocal causation, EES challenges Mayr’s ultimate/proximate causal dichotomy because it highlights how developmental changes alter selection pressures, and therefore how phenotypic changes can feed into genotypic changes. EES explains the bias towards functionality according to the relative reciprocal contributions made by the system’s various levels (gene regulatory networks, cells, organs, organisms, ecological groups, etc.) by studying the interactions between each level’s 1) subcomponents, 2) context, and 3) form (i.e. physical or mechanical constraints). For example, the changed behavior of an organism can be viewed as determined by subcomponents such as genes, cells, organs, systems; by its physical and social environment (which includes the ways that the organism changes each of these through interaction); and by the physical properties of the biological systems that push the organism towards certain changes but which can also be changed in turn (Lewontin and Levins 2007). It is acknowledged that these different contributions reciprocally determine one another as development proceeds so success in EES explanation will depend on the extent to which these different contributions can be integrated into a descriptive and predictive theory. Developmental bias is explained as either the result of past selection or as the emergence of a new functionality, but insofar as the latter plays a major role in integrating development at its various levels, the processes that ensure that there is a higher than random probability of functionality are poorly described.

This problem has been recognized clearly in niche construction theory, whose advocates have insisted that niche construction cannot be haphazard. Constructing niches is resource intensive and would not occur unless it was likely to increase fitness for the organism and/or its offspring. This is easily accomplished when the constructive activities have been selected for and therefore stereotyped (not unlike Dawkins’ ‘extended phenotype’ (Dawkins 1982), which is presented simply as an “externalized projection of the genotype” (Laland et al. 2014a, b)), but niche construction advocates insist that many niche construction activities and their broader consequences are often contingent and underdetermined. The implication is that novel niche constructing behaviour cannot be blind. In this vein, Laland and his colleagues note that the process must be “driven by the purposive (i.e. goal-seeking) activities of organisms in pursuit of their fitness goals” (p. 2416) but in the next sentence (and with scarequotes around the word ‘purpose’) they explain that these goals are survival and reproduction amidst the degrading forces of thermodynamics. And so, while they highlight the central role of the organism as purposive agent in the process, they avoid semiotic theory and fall back on adaptionist arguments. As a result, light is not shed onto how such goal-seeking activities actually lead to beneficial yet underdetermined niche constructions whilst Mayr’s proximal-distal causal dichotomy (which the authors here and elsewhere otherwise reject) is again perpetuated.^3^

The still-weak integration of teleology into otherwise promising EES explanations is also present in discussions of phenotypic accommodation.^4^ The two-legged goat, famous in developmental plasticity literature thanks to West-Eberhard (2003), rearranged its physiology and behaviour on multiple integrated levels to accommodate its loss of forelimbs, revealing the importance of novel organism-level coordinated response to developmental contingencies. West-Eberhard is quick to point out that organisms do this sort of thing with any novelty, regardless of whether it was produced environmentally, developmentally, genetically, or in some combination of these. However, she is much less clear about what is driving this wonderfully integrated accommodation. Without any clarification on what is motivating the organism, it is easy to lose sight of how West-Eberhard has foregrounded it as a causal nexus, and to revert back to genetic or environmental explanations (or combinations of these). Like niche construction, West-Eberhard’s two-legged goat puts the organism back in the centre of the developmental and evolutionary story, but it remains opaque with respect to how the organism converts the push and pull of various determinisms into causal efficacy. A biosemiotic approach fills in this explanatory deficit by insisting that the way in which the world appears to the goat is itself a necessary part of the capacity of the organism to function as a causal agent in development and evolution. From this view, it is only because something *appears* as desirable at a distance that the goat will put an effort into acquiring it, which drives the re-coordination of its anatomy. In other words, the meaningful and activity-eliciting Uexküllian *Umwelt* (von Uexküll 2010) that an organism constructs is both a cause and an effect of many of the phenotypic accommodations and niche constructing activities that are now receiving attention in EES theory.^5^ While the organism’s motivations to survive can be assumed and treated as background conditions by nonsemiotic theories, these motivations (and the environment as it appears to those so motivated) are themselves the product and process of the phenotype. Considering that EES analyses rely precisely on breaking down the assumption that background conditions operate independently of dependent variables (as we see in their challenge of the concept of a static selective environment, or static gene expression), evolutionary theory is incomplete insofar as it does not address the emergence, development, and contribution of the organism’s goal-based, meaning-making activities in the developmental process. And insofar as the phenotypic changes of a genetic intervention are among such developmental processes, an understanding and assessment of biotechnology is also incomplete without biosemiotics.

## The Contribution of Semiosis to Functional Ontogenesis

Biosemiotics attempts to understand and describe the process of meaning making in life: how it happens, what different types meaning-making occur, and what consequences the construction of meaning has. Causality is seen as triadic in semiotic systems (Houser and Kloesel 1992), consisting of a sign, an object, and an interpretant. According to Emmeche (2007), “[a] *sign* is anything that can stand for something (an *object*) in some interpreting system …, where “standing for” means “mediating a significant effect” (called the *interpretant*) upon that system (p. 94, italics in original). The interpretant includes all the effects that result through taking the sign as referring to the object. These maybe conceptual or emotional effects, but they may also be physically instantiated effects, and it is the latter which are observed in empirical biosemiotic investigations. For example, when an *E. coli* bacteria takes and artificial sweetener to stand for its food (see Stjernfelt 2007 for a discussion on this), this meaning making activity is evident in the physical interpretant that we observe: the bacteria swims towards the sweetener (it is also evident in the physiological changes that the bacteria undergoes). Prevalent amongst biosemioticians is the view that semiosis occurs and is mediated on multiple levels within biological systems, from the cell to the organism to the social system. Hence, the view presented is that even in simpler organisms, a strictly mechanical explanation (where an understanding of parts and their interactions allows sufficient and adequate modelling of the system) is deficient. Like niche construction and developmental plasticity theory, biosemiotics acknowledges the positive contribution that the organization and dynamics of the whole organism (and its whole sublevels and superlevels) play in the unfolding process. But unlike these approaches, it explicitly identifies the capacity of these systems to engage in the sort of triadic causality described here as a crucial dimension for understanding how these wholes make their positive contribution. Because the physical aspects of the interpretant are observable, biosemiotics does have an empirical research programme and is not merely a philosophical project. In particular, at least three important aspects differentiate semiotic interactions empirically from nonsemiotic ones. What shows up as significant is observable through 1) how the history and goals of the organism re-enter into and scaffold current semiosis (see Hoffmeyer 2015; Kull 2015), 2) how signification changes (i.e. learning) (ex. Kull 2015), and 3) through interpretive mistakes (as Stjernfelt’s (2007) *E. coli* mishap clearly shows). While semiosis is pervasive in living systems, it is still not widely acknowledged because most biologists assume it implies some sort of non-empirical neo-vitalism. It is, of course neither. But it does not insist that the only valid approach is to operate under mechanism as a heuristic methodology carried over from Newtonian mechanics. Biosemioticians critique the approach of biologists who continue to use communicational language to describe biological phenomena while insisting that such language is a convenient metaphor (Hoffmeyer 2008). This rationalization indicates a still persistent tendency to not consider the actual communicational interactions that are being modelled. A number of incoherent confusions result, among them the idea that ‘information’ is a physical substance that resides (for example) in the DNA, rather than something interpreted as such by organisms capable of drawing meaningful distinctions and enacting corresponding interpretants.

Nevertheless, it is not the case that semiotics will be able to explain all biological processes. Semiotic explanations are necessary but not sufficient for understanding biological phenomena, because organic systems are also physical and chemical systems and are therefore always subject to the constraints and possibilities that physicochemical phenomena affords on different scales (this can include mutations in the DNA as a result of physical factors, forms of “cellular noise” that arises from various stochastic influences, and the directive force of meso-level physical properties on cell form and dynamics as described in Newman (2012) and Forgacs and Newman (2005)). However, the biosemiotic view does suggests that these factors may not remain “purely physical” for long, as they become co-opted, transformed, and directed by the interpretive activities of organismic semiotic processes throughout development. But in any case, it is apparent that just as EES is incomplete without a semiotic understanding of how the organisms makes meaning of its world, biosemiotics is also incomplete without recognizing that semiosis takes place alongside and interpenetrating with other developmental resources. Indeed, everything from the reconstruction of the genome and epigenome to niche construction are semiotic processes. This acknowledgment is reflected in the way that many biosemioticians incorporate modern evolutionary and developmental theory into their work (ex. Hoffmeyer 2008; Bruni 2008; Sharov 2014; Kull 2015; also see introduction and special issue of *Biosemiotics,* “Semiosis of evolution” (Sharov et al. 2016)).

In fact, biosemiotics can help integrate views on plasticity in development with niche construction. While this has been partially recognized by traditionally non-biosemiotics scientists, (including Moczek (2015), Laland et al. (2008), Chiu and Gilbert (2015)), the most important integrating insights have been made by those in the community. In particular, the dynamic nature of semiosis (as opposed to the mechanism of computation and signalling) (Kull 2015), the concept of semiotic scaffolding (Hoffmeyer 2008, 2015), and multilevel semiosis (Bruni 2003, 2007) seem to particularly fruitful. The biosemiotic view recognizes that change in phenotype behaviour, form, and structure essentially implies plasticity and niche construction, but it casts these in ways that explicitly acknowledge and study their role within the goal-based meaning making activity of organisms at various semiotic levels. The effects of genetic interventions on phenotypes is, as we shall see, complexified by the biosemiotic observation that organisms engage in interpreting and reconstructing themselves in varied ways in response to such changes.

Developmental plasticity alters an organism’s ecological interactions, which leads to different sorts of niche construction. On the other hand, niche construction also influences developmental plasticity by changing the environment that the phenotype is accommodating. For these reasons, the inner organization and the outer organization of an organism are inherently connected (Laland et al. 2008). However, the point can be made more strongly as much of this “inner” plasticity is itself the outcome of various niche constructing semiotic activities occurring on the intercellular level (both by host cells and symbionts (Chiu and Gilbert 2015)). Laland et al. (2014a, 2014b) have suggested a strong “parallel” between internal and external constructive processes in development and evolution, while Moczek (2015) has discussed how niche construction can refer to the construction not only of external environments but internal environments as well, with the only difference being of scale. In fact, phenotypic accommodation, inner and outer niche construction always occur simultaneously because external and internal events are co-emergent and mutually implicated. Phenotypic accommodation entails niche construction, which is a way of framing the process by considering entities (such as cells, organisms, but also genes) in terms of their dynamic interactions with their developmental contexts. From a biosemiotic point of view, all this phenomena can be seen unified in the following sense. A semiotic entity (such as a cell or an organism) discloses a field of valence and significance through differentially responding to various things in its inner and outer environment in accordance with its goal-directed meaning making activities at any given time. These responses are interpretants that modify the relationship between that entity and that which appears as significant to it. The physical manifestation of the interpretant is the contribution that entity makes towards modifying itself and its relationship with its various environments. An interpretant has physical, and invariably semiotic effects widely distributed across the organism’s interactional domain. The process is called “niche construction” when we want to focus on how the living system is modifying external conditions around it. It is called “developmental plasticity” when we focus on how its internal conditions have been adjusted. But the division is somewhat arbitrary and depends on our taking a particular level in the hierarchy as our frame of reference and defining that which is internal and external to it.^6^ In both cases, we are in effect talking about the sort of “semiotic scaffolding” (Hoffmeyer 2008, 2015) that biosemiotic researchers recognize as integral to development, where the physical interpretants of past semiosis re-enter into the system as interactants and possibly significant objects in their own right. In this way, semiotic scaffolding is a general term that describes the contributions that the meaning-making semiotic elements of a living organism make in changing itself and its environment at the various levels at which this occurs.

## Biological Heterarchy and Semiotic Translation

In an analysis of genetic interventions, it is important to understand that any potential semiotic accommodations of the functionality of modified genetic material is nevertheless occurring in a system that has undergone a powerful initial disruption. In this section, I hope to show some ways in which the heterarchical nature of semiosis in organic systems suggests that genetic interventions may often be disturbing to system viability in a novel way. The argument is informed by insights into the nature of communication in heterarchical systems as described by Bateson (1979) and later by Bruni and Giorgi (Bruni 2003, 2008; Bruni and Giorgi 2015).

Genomes are within nuclei, which are within cells, which are within the organs of organisms that live in social and ecological systems. Life appears to be structured hierarchically, with organization nested within organization. But this hierarchy is not dictated by a unidirectional chain of command because each level is in recursive interaction with the systems within and outside of it. For this reason, McCulloch’s (1945) term “heterarchy” has been proposed as a better alternative when referring to the communicational organization of living systems (Harries-Jones 1995; Salthe 1993; Bruni 2003; Bruni and Giorgi 2015). A heterarchical system is one where interactants in a given level may reciprocally interact with other elements on that level but also with elements “above” or “below” it in the heterachy. Causality is therefore emergent and distributed between levels rather than unidirectional. However, while communication is bidirectional between levels, in heterarchies the nature of the communication is not symmetrical. Higher levels place boundary conditions upon lower levels in a way that does not normally occur in the other direction (unless the lower level systems have changed sufficiently enough to aggregate a significant difference (Bateson 1979)). As differences are aggregated into new differences, they can be seen as “translations” of information up levels in the heterarchy. And higher level changes translate information downwards through altering the boundary conditions of local interactions. The informational pathways up and down a heterarchically organized system occur through chains of translations (Bruni and Giorgi 2015). This translation process takes on a particular sort of pattern. As Bateson (1979) indicated (in his analysis of chains of logical typing that flip between descriptions and typologies of process), and Bruni (2003, 2008) developed in discussing molecular biology, these translations tend to occur through a process whereby digital codes on one level aggregate to form an analogical process on a higher level, that can in turn be interpreted digitally on that or some still higher level. For example, the binding of a receptor is a digital event insofar as it either happens or not, but the ongoing flux of information of all receptors in a cell would appear as an analog process to a semiotic system like the cell itself, which registers continuously varying magnitudes or states. Nevertheless, this meta-level aggregation may in turn get digitalized either by the cell itself that has response ‘thresholds’ or by other interacting systems on the cellular or the next higher level. And so on.^7^

An inserted transgene or edited gene can therefore affect semiotic processes at the genomic, the cellular, the tissue, the organ, the organism, the sociological (both human and nonhuman), and the ecological levels, and can also lead to various translations between these levels. Omic studies (such as transcriptomics or proteomics), which are the most comprehensive tests available on genetically modified organisms to date (Heinemann et al. (2011)), do not capture changes in semiotic activity within and between levels because they only present a decontextualized chemical inventory as a snapshot at a particular time. A “semiomic^8^“ (a term Hoffmeyer 2014 suggested) analysis would fail to capture the most essential aspect about semiosis: that interpretation is ongoing and contingently dependent on continuous organism/environment interactions at various levels and scales. This cannot be captured in a catalogue or inventory (Hoffmeyer would probably agree). Understanding the semiotic effects of genetic interventions is necessarily a temporal process, as it involves understanding how the system at its different levels interprets its relationship with the modified gene element(s) (and then its relationship with the interpretants it produces) during ongoing developmental and environmental vicissitudes. Alongside this complexity, such interpretations depend not only on the state of the organism and the particular situations it is faced with at a given time, but on the semiotic history of the organism as well.

Because heterarchical systems are sustained through step-wise translation up and down levels, often translating from digital to analog and then back again, threats to system viability are introduced when the logic of translation is not maintained. For Bateson (1979), it was crucial to acknowledge that system-sustaining communication generally occurs only within levels or between adjacent levels, so that lower level activity often needs to be responded to by the level *directly* higher up in order to be picked up by the level above that. Communication that “skips” levels is risky, a point which we shall see is relevant in the context of genetic interventions.

By definition, genetic engineering is an inter-level semiotic phenomenon. Moreover, it is one that involves translation that skips levels. This is because it employs meanings produced through human social life about “phenotypic traits” to intervene on the construction of meaning at the genetic level. The human meanings include not only the values and goals compelling the intervention, but also the conceptual frameworks modeling the genome, paradigmatic understandings about the nature of causality, information, phenotypes, mechanism, and related terms, the design interventions, and the evaluations of the consequences of the intervention.^9^ These understandings have obviously not been built up through digital-analog translations; the semiosis of the cell and the genome is translated directly into the sociohistorical interpretive system that we have available. A genetic intervention is produced, and it has systemic effects distributed across multiple organisms and across many reciprocally interacting semiotic levels. And we again interpret its effects without acknowledging the various translation processes that have gone on by the organism in response to the intervention. At each stage of the process, we deny that there is a multilevel interpretive system modulating and reworking the meaning of what we have done. Persistently ignoring this semiotic dimension means that we will continue making decisions as though such semiosis did not exist. For example, a transgene or edited gene may directly or indirectly lead to epigenetic, cellular, physiological, morphological, or behavioural changes. These in turn can be picked up as significant differences to other organisms interacting with the intervened upon organism, including humans, for whom beliefs and understandings about the “threat” or “safety” of modification might lead to differences in (for example) DNA methylation (Nikolova et al. 2014), neural circuits (Feder et al. 2009), endocrinal activity, an altered perceptual experience of environments containing “GMOs,” and changed decisions with respect to transgenic organisms. Differences “make” differences, getting passed around within levels and translated between them, as responses and responses to responses cycle through the system. While this is obviously true for many biological interactions outside of genetic engineering (raising a pet or hunting an animal also lead to semiotic cascades within and between systems), genetic interventions turn out to be “different” sorts of differences in important ways.

Some “different” differences with respect to genetic interventions are rooted in the nature of the multilevel semiotic interaction. As mentioned, Bateson (1979) insisted that the hierarchical nature of communicational systems would not function properly if communicational interactions occurred between non-adjacent levels. There are several reasons for this. The first problem concerns the correspondence between different levels of the system. For example, information gets translated as it moves up and down levels in the system and system viability seems to depend on progressive aggregations and responses to aggregations of information that occur as information moves ‘upwards’ in the system. If information skips a level “upwards” the concern is that small differences that may not normally warrant significant response may nevertheless lead to big changes, which can lead to exaggerated and therefore non-functional responses. Second, and relatedly, is the fact that the jumped intermediate level nevertheless still exists and is still semiotic, now interpreting and responding to changes that have occurred above it (and response to levels below it). In this example, oscillations in the system could be further exaggerated because the jumped level would interpret the higher-level information by adjusting accordingly, further validating the response.

It is therefore significant that genetic interventions are themselves communicational interactions that jump levels of mediation, translation and aggregation, and attempt to bypass the digital-analog translation chain that Bateson acknowledges (and that Bruni and Giorgi (Bruni 2008; Bruni and Giorgi 2015) develop). Before moving on, it is worth making clear how such genetic interventions differ from traditional breeding. With traditional breeding, symbolic ideas about breeding are on the same level as the interaction with the bred animals or plants. We intervene in breeding of phenotypes at the level of phenotypes. How these interactions get translated up and down the multilevel system is then determined by the endogenous and ecological semiotic processes within which the intervention takes place. It is for this reason that animals can be cross-fertilized in a way that combines dozens or even hundreds of allele variants with very little risk of debilitating consequences for the offspring (whereas transgenic interventions often lead to aborted embryos and serious deformations even though they typically involve the addition of very little genetic novelty). New traits in non-transgenic organisms are system-level occurrences that emerge through interactions at and between multiple spatial and temporal scales. The ‘result’ is the phenotypic form, which is the appearance of these integrative developmental processes evident to humans observing the organisms. It is not an ‘idea’ taken from one context (the idea that a trait can be moved between species, for example, where the notion of an isolated trait is a product of human cultural organism-level semiosis), reified in a second context (the molecular biology of another organism’s DNA, which is now treated as though it were the cause of the trait), and then imposed into a third (the transgenic organism’s developmental process). The “information” in the DNA code is given to it by the interpretive and reinterpretive activities of the cell, which is also always generating information about nongenetic intracellular elements as well as extracellular information. Much of this extracellular information may itself be a translation of the organism’s outer environment by the multicellular sensory and perceptual system transducing environmental signals. The context backgrounding any difference is set by the analog flux of elements across and between levels that co-inform the cell. While the cell may ‘digitalize’ information when it transcribes particular parts of the DNA, it does not digitalize in the same way that we do when we parcel a chunk of code and assign it a specific role in the downstream phenotype. This is because on our phenotypic level as science-constructing beings, our digitalization of information in the code is informed by an entirely different set of analog elements. It is difficult to see how incommensurability could be avoided but a good start would be to at least try and understand these varied translation processes. A heterarchical view would recognize that the information in DNA is directly generated by the cell, which is itself co-informed by other elements in a multilevel system with semiotic causality distributed throughout. In such a view, “information” is a transient and enacted process, the outcome of interpreting systems awash with contingent fluxes of differences to which it is always contextually dependent. Ignoring this assumes that the semiotic constructs we establish in one domain of interactions can be applied to another (if not ontologically then at least pragmatically (Barnes and Dupré 2008)). We continue to use the short hand idea that there is a gene “for some phenotypic trait X” despite the fact that this idea emerged at a certain historical stage of scientific inquiry and with certain presumptions about the nature of causality and communication that are now untenable (Keller 2000; Noble 2006).

## The Semiosis of “Side Effects”

The argument suggested in this section is that even though genetic interventions represent a new type of disturbance to organisms, as suggested by their heterarchical organization in the previous section, organisms nevertheless try to make sense of these disturbances and potentially co-opt or functionally transform the interventions they undergo. In other words, in the language introduced early in this paper, we should expect that development is biased towards putting modified genes towards functional use. Indeed, we do have evolutionary evidence that foreign genes have been deployed adaptively as evidenced by their ubiquitous presence amongst diverse species (see, for example Crisp et al. 2015) - though obviously there will be no such evidence for cases where organisms were unable to accommodate the novelty. To what extent organisms can bias genetic modification towards functionality successfully is an open empirical question to be examined on a case by case basis, and on various biological scales from the cell to the ecosystem, but the possibility that “side effects” are partially interpretants has important consequences for how we view genetic modification.

While the conventional view sees transgene insertions and gene-editing from an informational perspective as inherently unproblematic –as simply the addition of a new source of variability into an evolving stochastic system- some critics argue that the cellular reorganization of genomic information implies a system-level organization that is compromised through genetic engineering. For example, Ho (2014) insists that because cell-directed genome changes imply a functional role for transposons and other DNA reconstructing elements, the adaptive capacity of the cell is jeopardized by the insertion of genes into the system. The network effect of these genes is unknown. Through complex pleiotropic interactions, genetic interventions are seen to compromise the adaptive organization of the cell. While such unintended effects are certainly possible, such a view is not necessarily nor universally implied by advocates of cell-led genome reorganization (such as Shapiro 2011). System-level organization is also likely to sometimes ensure that the transgene or edited gene is biased in its developmental expression, subordinated by the teleological activity of the cell by the sort of semiotically-based activity introduced in this paper. From this perspective, Ho risks reaffirming the very gene-centric view she critiques in genetic engineers (albeit a relational gene-centrism) because she places predominant emphasis on the causal consequences of inserting genes into systems instead of the nonrandom semiotic reorganization of system elements in a temporal sequence of biological activity that is implied by a semiotic perspective. Shapiro’s (2011) point is more consonant with a view that hypothesizes the organism as a causal locus adaptively reorganizing both its external and internal relations (which are also in close correspondence) to ongoing threats and affordances (ex. West-Eberhard 2003). Though West-Eberhard only makes the point with respect to epigenetic alterations of the genome, it is easy to see that the genome is itself an ‘inner environment’ to be accommodated to but also reorganized, a niche to be constructed like any other (Odling-Smee et al. 2003). When considering genetic interventions, it is therefore sufficient neither to undermine the importance of systemic effects nor to hold a priori that all unexpected consequences are the result of disrupting existing semiotic processes on varied levels. In different ways, each of these approaches still imposes a dyadic logic onto the organism. Those who hold to the importance of ‘screening’ a transgenic or gene-edited organism through short term tests fail to acknowledge the extent to which the informational value of the genetic modification is reconstructed in various ways throughout typical and atypical ontogenesis. On the other hand, those who argue that ‘side effects’ are systemic ‘disruptions’ also underappreciate the role that semiosis plays in the various changes that an organisms develops as a result of a genetic intervention.

Of course, not all “side effects” are the result of semiotic interpretations on the part of the system. When a transgene is placed in the middle of an important regulatory gene, leading to immediately fatal or severely debilitating effects, such consequences are obviously the result of shutting down organismic functions rather than any organismal functional response to the transgene. The “side effect” in this case is, in part, the disruption of semiosis. Such a distinction is important, showing that it should be possible to classify categories of effects according to whether the response is semiotically integrative, disruptive, or a mixture of both. A semiotic analysis of these side effects may be usefully developed through a consideration of the phenomenon of ‘generative entrenchment’ (Wimsatt 1986, 2001). According to Wimsatt, the likelihood that a change in some developmental element will be functional (and adaptive) is inversely correlated with the likelihood that that element will have subsequently occurring developmental elements dependent upon it. This means that those elements that appear early on during ontogeny are those less likely to be modifiable through either developmental plasticity or genetic evolution. The semiotic activity of the system at its various stages of development also may rely in different ways on characters being locked-in early on. The implication is that novel traits are more likely to be less strongly associated and integrated into other traits, more likely to appear later during ontogeny, and therefore relatively more plastic in the face of environmental contingency. This is problematic given the commercial and safety standardization requirements placed on transgenic and gene-edited organisms. The significance of this point will be discussed at the end of this section. Locking in unproven new traits is simply not the way that evolution generally proceeds because, before getting entrenched, a trait has to show its value in a variety of ecological and physiological circumstances and dynamic relationships, but also because it needs to have essential subsequent developmental stages dependent upon and integrated with it. This is likely why (in part) developmental plasticity “tries out” new traits before their getting assimilated genetically, a process which some argue is even more consequential than evolution led by genetic mutation (West-Eberhard 2003, 2005).

It is necessary to generate a semiotic understanding of the ‘side effects’ of genetic interventions to gain clarity with respect to how and when given changes are the result of integrative interpretation rather than consequences of disturbance. This is likely not a simple task because whether something is functioning as a sign, an interpretant, −or both, or neither-, often depends on contingent factors such as timing, but also on the level of analysis in the heterarchical system. For example, the physical consequences of semiotic activity on one level may or may not get picked up as significant on either or lower levels. Moreover, a novelty can initially disrupt semiosis but at some point get accommodated by it, indicating a dynamic temporal system. Finally, so far as interpretants manifest physically, aspects of them can be taken subsequently taken up as signs, such that what is significant about the circumstance can be different for two entities interacting within this changed context (or even for the same entity interacting with that physical consequence at two different times).

The semiotic co-opting of genetic interventions can be observed in several different ways. It may occur through the cell altering the modified gene’s expression directly. Transgene silencing through methylation would be a simple example of a way in which the organism generates the informational value of the transgene by directly reconstructing it, but upregulation, downregulation, selective expression, chromatin restructuring, cell-mediated DNA editing such as exonization and intronization, and intragenomic movement of the vector are other examples. Whether or not the cellular interpretation of an edited gene or transgene physically alters the induced modification depends on a variety of factors, including the nature of the modification itself, its genomic location, the presence of promoters or other vector elements that ensure expression stability by preventing cellular adjustments and coordinations of function, the immediately ongoing environmental context of the cell and organism, and the history of the cell and organism.

If the modified gene’s expression is not directly altered by the cell, the modified gene can nevertheless get integrated into the system through alterations made elsewhere in the system. The gene may express itself according to what the scientist hoped while other genes shift their own expression patterns. Behaviour at the cellular, organismic and ecological levels may also semiotically accommodate the novel gene expression.^10^ A modified gene can be interpreted in a certain way by the cell, which is in turn interpreted by the tissue or organ it is a part of, which are in turn interpreted by that same cell or by others. Reciprocal interactions between levels occur through time. While it is possible to say (from a dyadic point of view) that the cascades of changes throughout the system are all the ‘effect’ of the modified gene, it is more accurate to recognize that causality is distributed throughout the system (Oyama 2000; Noble 2013), and that the efficient causality of this or that component is often constrained by boundary conditions set up through semiotic interactions at different system levels.

On the other hand, one should not be hasty in assuming that multilevel integrated changes imply that semiosis is occurring at the organismic level (for example, the integration of plant trophism does not necessarily indicate that the plant functions semiotically as a unit. Further analysis of whole-organism semiosis would be required to establish this (Affifi 2013)). In any “viable” genetically modified organism, semiosis often may be occurring at different times and scales across its multilevel organization. So, for instance, the upregulation of another gene in response to the presence of a modified gene could initially be a disruptive side effect based on proximity but one that *then* gets integrated into a gene regulatory network that ends up as somehow functional. Whether or not a “side effect” is actually the interpretant of the organism’s semiotic activity is important in helping us understand the health and autonomy of the organism, and the nature of context-dependent information. It is a comprehensive shift away from the dyadic conception which refuses the possibility that responsive teleological processes direct the fate of genetic modification in development. This has considerable consequences for the semiosis of genetic interventions at the human sociocultural level (i.e. how we understand what is occurring in a transgenic organism) insofar as it challenges certain hubristic conceptions of the place of humans in nature (see below).

However, even if such co-opting accommodations are widespread, whether or not the genetic intervention is “safe” is still an open question. Our capacity to assess the impacts of a given genetic intervention depends in part on our ability to distinguish between cases where the occurring change represents a disruption or loss of function, cases where the organism is semiotically accommodating the intervention through co-opting it, and cases where the impacts are semiotic disruptions. How we begin to discriminate and analyze these circumstances will dictate what course we take with respect to engineering the biosphere in the coming century.

Many of the same questions re-occur when considering the “ecological side effects” of genetically modified organisms in the wild. Consider the case of an “invasive species.” It is not always clear at what point the spread of a novel species shifts from being a disturbance to a healthy eco-semiotic readjustment. For example, in a recent study it was observed that many other species “piggybacked” on the introduction of honeysuckle into Pennsylvania woodlands. The honeysuckle increased the fruit-eating bird population, which then helped spread the seeds of indigenous fruit trees too (Gleditsch and Carlo 2010). On the other hand, it has been long noted that invasive species are often those that reclaim disturbed land and often transform it into habitats that facilitate the eventual succession of an ecological community. Whether or not a genetically modified organism’s ecological behaviour is considered a disturbance or a multispecies semiotic accommodation and niche reconstruction needs to be decided with some care and rigour and the semiosis of the transitions from one to the other (and at various time scales and organismic levels) needs to be better articulated. While it would be reckless to assume that the larger system is “intelligent” enough to accommodate and co-opt *any* perturbation, it is also shortsighted to think that there is no system-level reworking of the communicational dynamics and interrelations between its component members. We currently lack the language and the methodologies to separate these types of cases at the various levels in which they can occur, from cell behaviour to ecological communities.

Earlier, I raised the point that minimizing “side effects” likely has side effects of its own. I want to get back to this point briefly before moving on. Insofar as a transgene has an effective promoter, a stable genomic locus, and a stable environmental context, it can be treated as more or less predictable, and the semiotic activity of the system is hidden behind the appearance of mechanism. The insertion of viral promotors, transgene duplicates, and the ecological standardization of industrial agricultural practices are all examples of techniques that reduce interactive contingencies. What this often means, is that these mechanisms also reduce or regulate the semiotic activity of the system. Much research is devoted to attempting to minimize the instability of the expression and behaviour of modified genetic elements (ex. Butaye et al. 2005; Kohli et al. 2004). To this end, the apparent safety of transgenic and gene edited organisms hinges on their capacity to behave reliably, which is itself contrary to the conditions for systemic health and its requirement of diverse semiotic responsiveness. Industry seems to be caught in a difficult situation because the patentability, marketability, and assurance of health and environmental safety (at least in the short-term) all seem to depend on the product behaving in a predictable way that is at ends with the general emergence of novelty in evolving systems.

## The Level of Human Symbolic Semiosis

Both our attempts to control the semiotic activity of transgenic organisms and their subversions of this control feed back into the human conceptual domain. These cannot be dealt with satisfactorily in a paper of this length but can be hinted at here. Genetic engineering leads to certain ways of thinking about ourselves in relation to other species, which propagate in our conceptual/material ecosystems in various ways and so make up part of the environment to which we must thereafter accommodate. And just like an organism modifying the splicing or epigenetics of a given gene, we also modify our understanding of concepts in order to accommodate novelty in our mental ecologies. In this final section, I will conclude by outlining some of the ways in which genetic engineering affects our mental ecology and how we accommodate these new ways of thinking for better or worse. Many of the ways we conceptualize genetic engineering influence whether and how we engage in genetic interventions, so this is yet another example of how diverse semiotic levels have effects upon one another. I will then describe ways in which we might better think about genetic engineering in order to avoid some of the undesirable effects of these accommodations.

This paper has developed a different conception of unintended “side effects” than that offered by both proponents and critics of genetic engineering technologies. The concept of an unintended side effect conventionally suggests the notion of a system that has lost some of its integrity rather than one responsively exemplifying its dynamic integrative capacity. The “actor” according to this conventional view is seen as the scientist who made the DNA insertion. From the point of view suggested in current evolutionary biology and made explicit by semiotics, “side effects” can sometimes be seen as ways in which the organism actively readjusts itself to a systemic disruption, displacing the human as the sole actor in the system. Of course, this does not mean that “side effects” are necessarily beneficial to the organism (addiction involves semiotically driven ontogenesis, for example), and it certainly does not mean that these changes are beneficial to us or other interactants engaging with the modified organism. The point is simply that as long as we recognize only ourselves as the primary causal agents in the informational nature of the DNA code, and seek to minimize “side effects” by building in constraints that prevent the dynamic semiotic activity of genetically modified organisms to their changed conditions, we perpetuate unwarranted anthropocentric attitudes and behaviours.

Reinforcing the illusion that unidirectional mechanistic approaches are appropriate for engineering living organisms may lead to the assumption that such an operating ontology is also appropriate in other biological contexts. Ideas function metaphorically in mental ecologies and get applied (so say Peirce and Bateson (1979)) abductively to situations that appear analogous. If genetic engineering has led to multiple successes based on the assumption that organisms are giant machines programmed by genes, it becomes a lot easier for us to believe that organisms can and ought to be treated as such. Ultimately, the mechanistic treatment of human beings is also at issue. Such a process also risks reifying reductionism as an explanatory strategy. While the apparently isolated gene operating as a unit in phenotypic development is actually a construct dependent upon maintaining certain conditions so as to prevent inadvertent pleiotropy, epistasis, alternative splicing, nucleotide rearrangements, or any of a number of other semiotic activities at one or various organismal levels, the fact that only stable genetically modified organisms are sought after and marketed creates the illusion that genes normally behave as minute and individuated causal entities. This leads to the likelihood that we will continue looking for atomistic, bottom-up genetic explanations and interventions.

Further, the fact that the source of creativity in the biosphere is concentrated in human activity (which used to be more narrowly limited to the reconstruction of inanimate things) means that the ecological and evolutionary processes will come to be increasingly seen as something to be steered by humans. While it is unlikely that we will ever get to a point where it convincingly appears that we are able to guide such a massively complex process, the notion that we are headed that way may well inject a certain hubris into the way in which we interact in the biosphere. Engineering ecosystems often treats organisms non-semiotically and reproduces the same errors as engineering the genome. Each lacks sensitivity or appreciation of the fact that ecologies proceeds through distributed causality at multiple spatial and temporal scales, many of which are open-ended, interpretive, and cross-communicating. While the consequences of assuming that life is not semiotic may be self-correcting (insofar as they may lead to system failures that shake humility back into our operating assumptions) it is usually the case that humans can minimize the amount of epistemological reorientation required of their habitual modes of engagement by limiting their perceived error to certain particular situations (*this* particular genetic intervention was problematic because X), which localizes and partitions the humility required in the face of multilevel semiotic systems.

I suggest that we re-examine genetic intervention more closely in order to understand the semiotic activity that results from it through various levels of biological activity. The extent and nature of “side effects” need to be understood and discussed widely, and should clearly be integrated into school biology curricula. Unfortunately, our understanding of the developmental and evolutionary logic of semiotic process is itself still in its infancy, and so our capacity to grasp the nature and consequences of genetic interventions will for now remain naive. For this reason alone, we ought to exercise caution, at least until triadic and heterarchical logic is something we are comfortable with and can rely on in order to decide the advisable scope such technologies should play in society.

## Footnotes

1 “Development bias” is a term given for those nonrandom structural or behavioural novelties that emerge during development. Because phenotypes develop in such biased ways, natural selection is not working with blind variation (Arthur 2004). A similar, lesser known term, “regulative variation” has been proposed in the context of biosemiotics (Sharov 2014).

2 Developmental systems theory (DST), with its focus on the active construction of information (genetic and otherwise) by the organism throughout development, could be added to this list (Oyama 2000; Oyama et al. 2001)

3 As Juarrero (1999) has persuasively shown, the notion that an organism can be an active, intentional being with causal potency was observed as incompatible with a mechanical conception of the universe driven by efficient causality. However, according to this author, the circular causality between parts and wholes that occur in “complex adaptive systems” such as lifeforms insures that the activity of the whole (and therefore teleology) is once again a valid part of scientific explanation. Biosemiotic theories claim that semiosis are organismic activities with just this sort of causal potency within such circular systems.

4 And synonymously, phenotypic plasticity, developmental plasticity, ontogenetic plasticity, and associated terms.

5 Concepts such as plasticity and niche construction also shed light on our understanding of Umwelt. The concept of an Umwelt as a catalogue-able species-specific field of values and significations disclosed by the active organism, is called into question by these developmental theories through their recognition of radically contingent and novel form and behaviour during ontogenesis.

6 Biosemiotics can also deal with the ambiguity in the term “construction.” Many biologists take a mechanist approach to understanding construction, eschewing the lurking phenomenological sense even though it was made explicit by early niche constructionist, Richard Lewontin (1983) (and is the “ontological” sense of construction that is criticized by Godfrey-Smith 1996). Lewontin was much clearer about how the organism qua organism contributes to developmental plasticity as a causal agent. He realized that the organism’s ability to construct its external world and the way in which the organism constructs what is relevant in its environment, were inherently connected. Lewontin wrote that “the environment is not a structure imposed on living beings from the outside but is in fact a creation of those beings. The environment is not an autonomous process but a reflection of the biology of the species” (99). Lewontin’s concept of “environment” here is clearly consonant with the biosemiotic view, and he was concerned with how an organism’s environment and its external world interact. He insisted that “[o]rganisms alter the external world as it becomes part of their environments” (64), that that which appears to an organism and affords the organism’s activities is that which gets modified directly and so is also that part of the external world that undergoes change.

7 Some evo-devo biologists are observing cellular dynamics in ways that are converging on this Batesonian insight. For example, Moczek (2015: 1) has recently described how niche construction can be used to make sense of gene regulation in the following way: whatever transcriptional and translational events take place in a given cell are influenced by the specific cellular environments that cell finds itself in, characterized for instance by the presence or absence of key resources such as microRNAs, transcription factors, nutrients, signals from neighboring cells, and other signatures of past developmental events. While many of these components of the nuclear and cellular environment involved the earlier expression of specific genes, at any given developmental time they collectively make up an environment that defines the developmental context within which a given cell finds itself and to which it responds by altering its transcriptional or translational activity, thereby again shaping the environmental conditions in which the same cell will find itself later in development.

8 Hoffmeyer defines the semiome as “the entirety of an organism’s semiotic tool set: i.e., the means by which the organisms of this species may extract significantly meaningful content from their surroundings and engage in intra or interspecific communicative behavior” (Hoffmeyer 2014: 11).

9 This is because it involves symbolic ideas generated through human culture about gene-phenotype relations, and the altering of genes based on those ideas. Such symbolic ideas include theories about where and how information is located in the genome, which are a part of the organism-level semiotic activity of the human phenotype emerging at the aggregate level of the CNS/body in interaction with its environment. But genetic interventions rely on such ideas about the nature of information apply to create a difference in a semiotic system that does not respond to construct information in the predictable and spatially well-defined ways that are assumed in the symbolic description inherent in altering parcels of DNA code.

10 Some of these systemic effects may be due to the invasive nature of the intervention process itself. In addition to the unintended consequences of the transgene itself, transgenic interventions also stress plants through various tissue handling and regeneration processes (Filipecki and Malepszy 2006), leading to effects that may or may not be semiotic in nature.
